# Qualitative Phytochemical Profiling and Antimicrobial Activity of *Viscum album* L. Extracts Obtained by Microwave-Assisted Extraction: Influence of Milling Pretreatment

**DOI:** 10.3390/plants15213311

**Published:** 2026-10-26

**Authors:** Dušica P. Ilić, Ljiljana P. Stanojević, Dragan Z. Troter, Sandra S. Konstantinović, Jelena B. Zvezdanović, Bojana R. Danilović, Ivan S. Ristić

**Affiliations:** 1 Faculty of Technology, University of Niš, Bulevar Oslobođenja 124, 16000 Leskovac, Serbia; stanojevic@tf.ni.ac.rs (L.P.S.); dragant@tf.ni.ac.rs (D.Z.T.); sandra@tf.ni.ac.rs (S.S.K.); jzvezdanovic@tf.ni.ac.rs (J.B.Z.); bojana.danilovic@tf.ni.ac.rs (B.R.D.); 2 Faculty of Technology, University of Novi Sad, 21102 Novi Sad, Serbia; ivan.ristic@uns.ac.rs

**Keywords:** *Viscum album* L., microwave-assisted extraction, milling pretreatment, qualitative UHPLC-DAD-MS/MS profiling, antimicrobial activity, total extractive matter

## Abstract

This study compared a defined milling pretreatment with a non-milling preparation of *Viscum album* L. plant material subjected to microwave-assisted extraction (MAE) with 70% (*v*/*v*) aqueous ethanol under identical operating conditions. The milled material was processed for 30 s using a laboratory mill, whereas the non-milled material was cut into smaller fragments. Total extractive matter (TEM) increased with time and reached the maximum after 20 min, with values of 5.52 and 5.01 g/100 g plant material for milled and non-milled samples, respectively. Qualitative UHPLC-DAD-MS/MS analysis revealed that the most dominant compounds in both extracts are organic acids, phenolic acids and flavonoid glycosides, followed by chlorophyll derivatives, coumarin glucosides and sesquiterpenoids. Both extracts demonstrated antimicrobial potential against *Bacillus cereus*, *Bacillus subtilis*, *Staphylococcus aureus*, *Escherichia coli*, *Klebsiella pneumoniae*, *Proteus vulgaris*, *Pseudomonas aeruginosa* and *Candida albicans*. The MAE process that used the milled plant material had lower specific energy consumption, referred to as the TEM amount, together with the overall process cost. According to the differences in TEM yield, qualitative phytochemical profile and antimicrobial activity of the extracts, the milled *Viscum album* L. plant material is recommended to be used for future MAE optimization experiments in order to obtain extracts that can have different applications.

## 1. Introduction

The extraction of bioactive compounds from medicinal plants depends on plant characteristics, sample preparation, and extraction conditions. Previous research has demonstrated antimicrobial activity in extracts obtained from different organs of *Viscum album* L. [1], while plant-derived natural products remain important sources of compounds for biological and pharmaceutical investigations [2]. Improving extraction performance and characterizing the resulting extracts are therefore relevant objectives in medicinal-plant research. *Viscum album* L. (European mistletoe) contains diverse constituents with reported biological properties [3,4,5]. Its phytochemical composition may vary according to the host species and environmental or harvesting conditions [6,7]. Chromatographic studies have further demonstrated the complexity of mistletoe preparations [8]. Reported activities include antimicrobial, anti-inflammatory, and antioxidant effects [9,10,11,12]. Liquid chromatography coupled with tandem mass spectrometry has been used for detailed characterization of *V. album* metabolites [13]. Other investigations have examined the biological effects of viscotoxins and additional mistletoe constituents in experimental cellular and preclinical models [14,15,16]. These findings underline the importance of considering phytochemical composition when evaluating mistletoe extracts, without assuming a direct relationship between individual compounds and observed antimicrobial effects.

Extraction efficiency depends on the plant matrix and extraction method [17]. Microwave-assisted extraction (MAE) has been investigated as an alternative to conventional extraction methods because it can facilitate mass transfer and reduce extraction time [18]. Efficient use of extraction resources is also an important consideration in green extraction approaches [19], while solvent composition, temperature, extraction time, and material preparation can affect the recovery of plant constituents [20]. Mechanical pretreatment, including milling, may modify matrix accessibility and influence extraction behavior. However, its effects should be further evaluated under defined experimental conditions.

Although the phytochemical composition and biological activities of *V. album* have been studied extensively, the integrated comparison of milled and non-milled material under identical MAE conditions remains insufficiently explored. The present study therefore compared these two preparation procedures using *V. album* collected from *Malus domestica* Borkh. in the Stara Planina region of Serbia. Extraction was performed with 70% (*v*/*v*) aqueous ethanol. The objectives were to compare total extractive matter (TEM), characterize the extracts by qualitative UHPLC-DAD-MS/MS analysis and evaluate antibacterial and anticandidal activities. In addition, an initial framework regarding the techno-economic analysis and evaluation of the energy-related indicators was provided. This study combines assessments of extraction performance, qualitative phytochemical profiles, antimicrobial responses, and energy-related indicators within a common experimental framework.

## 2. Materials and Methods

### 2.1. Plant Material Preparation

*Viscum album* L. plant material (fresh leaves and young stems, without berries) was collected from *Malus domestica* Borkh. in the Stara Planina mountain region, Serbia, at the Babin Zub locality (43°22′08″ N, 22°36′30″ E; 1758 m a.s.l.) in January 2023 (batch L160123M). The plant material consisted of fresh leaves (60%, *w*/*w*) and young stems (40%, *w*/*w*), without berries, corresponding to a leaf-to-stem mass ratio of 3:2. Leaves and young stems were used as a mixture rather than analyzed separately. A voucher specimen (No. 3655 HFF) was deposited in the Herbarium of the Faculty of Pharmacy, University of Belgrade, Serbia. The taxonomic identity of the plant material was additionally supported by published data for *V. album* from Serbia [21]. The plant material was prepared in two forms: sample I (milled material) and sample II (non-milled material). Sample I was milled for approximately 30 s using a laboratory mill (IKA A11 basic, Staufen, Germany) and then sieved through a 0.5 mm sieve to obtain a defined particle-size fraction. The laboratory mill (IKA A11 basic, Staufen, Germany) was operated at a single fixed speed. Sample II was not milled but was cut into 1–2 mm fragments using stainless-steel laboratory scissors, with fragment dimensions checked using a vernier caliper (Bochem Instrumente GmbH, Weilburg, Germany). A total of 3300 g of plant material was prepared for each treatment: milling (Sample I) and cutting without milling (Sample II).

Both samples were stored separately in sealed dark-glass bottles at 5 °C, protected from light and moisture, until analysis. Moisture content was determined by oven drying at 105 °C. The values obtained after 60 and 120 min were 9.56 ± 0.11% and 10.05 ± 0.03%, respectively, for sample I, and 10.22 ± 0.11% and 10.95 ± 0.03%, respectively, for sample II; no further change was observed after 120 min. The 120 min drying period was selected because moisture values changed between 60 and 120 min but remained stable after 120 min.

### 2.2. Chemicals and Reagents

For UHPLC-DAD-MS/MS analysis, purified distilled water and methanol (LC/MS grade; Fisher Scientific Co., Waltham, MA, USA) were used. Formic acid (HPLC grade) was obtained from Carlo Erba (Val-de-Reuil, France). All reference standards (apigenin, caffeic acid, chlorogenic acid, citric acid, hyperoside, isoquercitrin, kaempferol, luteolin, malic acid, neochlorogenic acid, quercetin, rosmarinic acid, rutin and tiliroside) were from Sigma-Aldrich (St. Louis, MO, USA).

### 2.3. Microwave-Assisted Extraction (MAE)

For each extraction, 100 g of *V. album* L. plant material (milled or non-milled) was combined with 1000 mL of freshly prepared 70% (*v*/*v*) aqueous ethanol in a round-bottom, one-necked glass flask, corresponding to a liquid-to-solid ratio of 10 mL/g (1:10, *w*/*v*). A 70% (*v*/*v*) aqueous ethanol solution was selected because aqueous ethanol is commonly used in conventional extraction procedures. The solvent composition was kept constant throughout the experiments to enable comparison of milled and non-milled plant material under identical extraction conditions.

After fitting the condenser, the extraction system was placed in a laboratory microwave extraction unit (Milestone ETHOS EZ, Milestone Srl, Sorisole, Italy). MAE was performed for 2, 4, 6, 8, 10, 12, 14, 16, 18, 20, or 30 min at 60 °C, using an operating frequency of 2.45 GHz and an irradiation power of 500 W. Temperature was monitored using the infrared temperature-monitoring system integrated in the microwave unit and maintained at 60 °C. After extraction, the mixtures were cooled to room temperature (20 °C) and centrifuged in a TH16B centrifuge (Hong Kong, China) at 3000 rpm for 15 min. The liquid phase was decanted, subjected to a second centrifugation under the same conditions, and filtered through sterile gauze to remove residual fine particles. Ethanol was removed under reduced pressure using a rotary evaporator (Hei-VAP, Heidolph, Schwabach, Germany) at 50 °C, after which the extracts were dried in a vacuum oven at 40 °C to constant mass. The resulting extracts were stored at 4 °C until analysis. All extractions were performed in triplicate.

### 2.4. Determination of Total Extractive Matter (TEM)

Total extractive matter (TEM) values were determined gravimetrically for extracts obtained after 2, 4, 6, 8, 10, 12, 14, 16, 18, 20, and 30 min of extraction by drying to constant mass at 105 °C. TEM values were expressed as g of extract per 100 g of initial plant material (as-is basis). TEM yield values were expressed as g of extract per g of initial plant material (as-is basis). The quantity of TEM extracted per batch (in g) was calculated by the following equation:

*TEM extracted per batch* = *TEM yield* · *m (dry plant material)* (1)

where m (dry plant material) is the mass of the solid plant material without moisture (after drying). For comparison, the TEM values at each extraction time were expressed as a percentage of the highest overall TEM value observed across both plant-material preparations under the investigated conditions, according to Equation (2):

*R* = 100 · *A*_1_/*A* (2)

where R is the percentage ratio (%), A_1_ is the TEM value obtained at a given extraction time, and A is the highest overall TEM value observed across both plant-material preparations under the investigated conditions (5.52 g/100 g plant material). Thus, the denominator was common to both the milled and non-milled samples.

### 2.5. Qualitative Phytochemical Profiling by UHPLC-DAD-MS/MS

**Figure 1:**
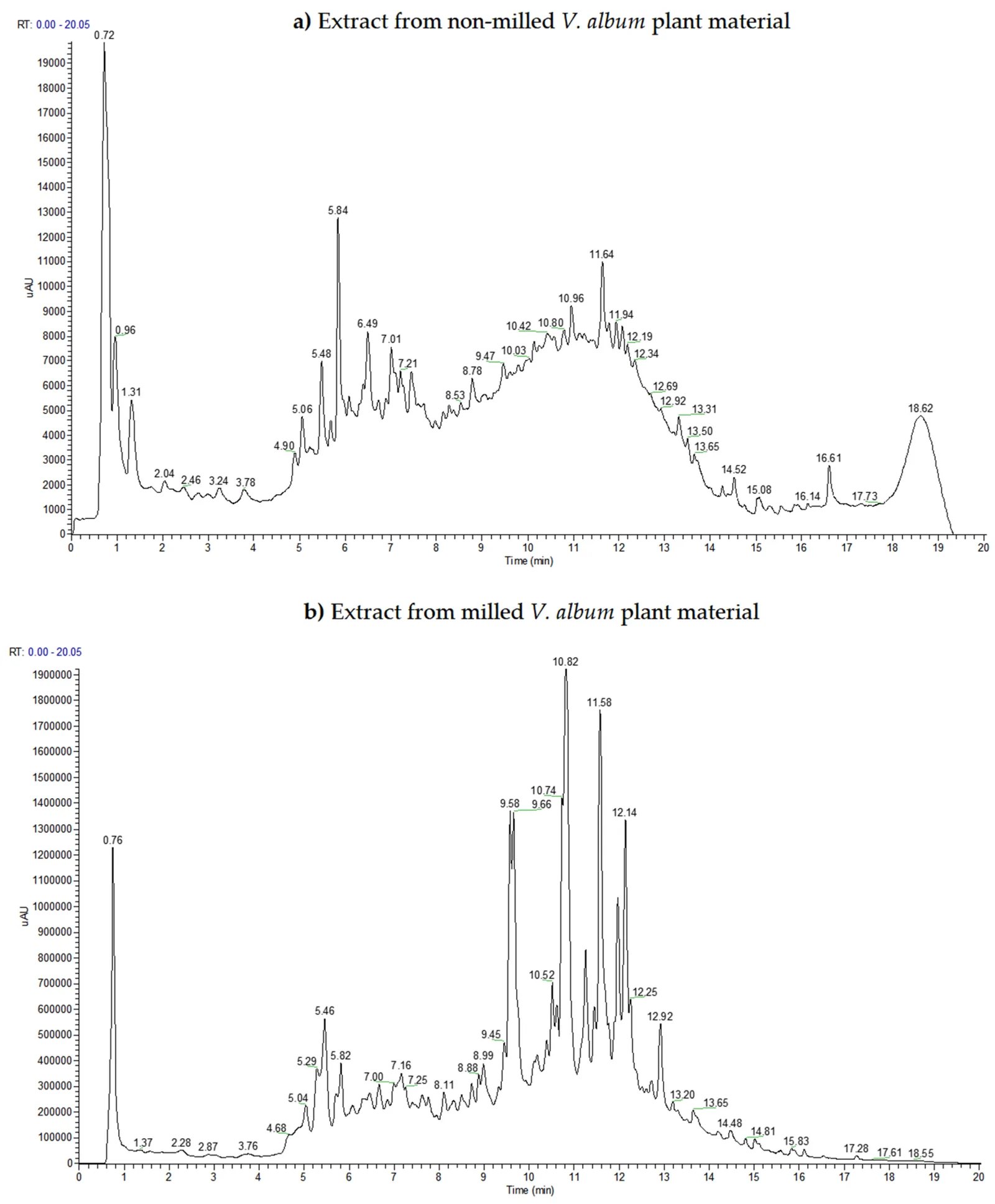
Representative ultra-high-performance liquid chromatography with diode-array detection (UHPLC-DAD) chromatograms of extracts from non-milled (**a**) and milled (**b**) *V. album* plant material recorded at 280 nm.

**Figure 2:**
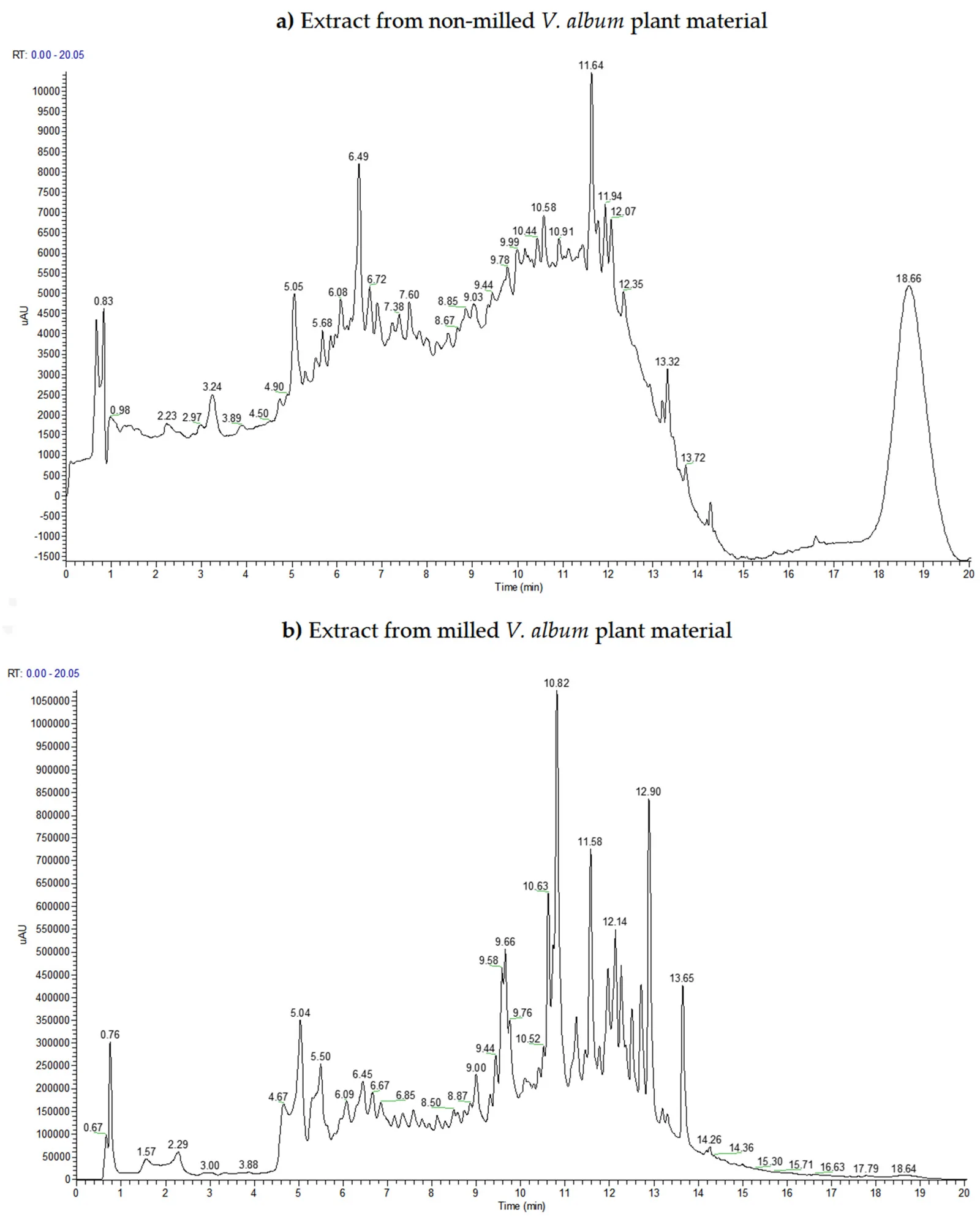
Representative UHPLC-DAD chromatograms of extracts from non-milled (**a**) and milled (**b**) *V. album* plant material recorded at 330 nm.

**Figure 3:**
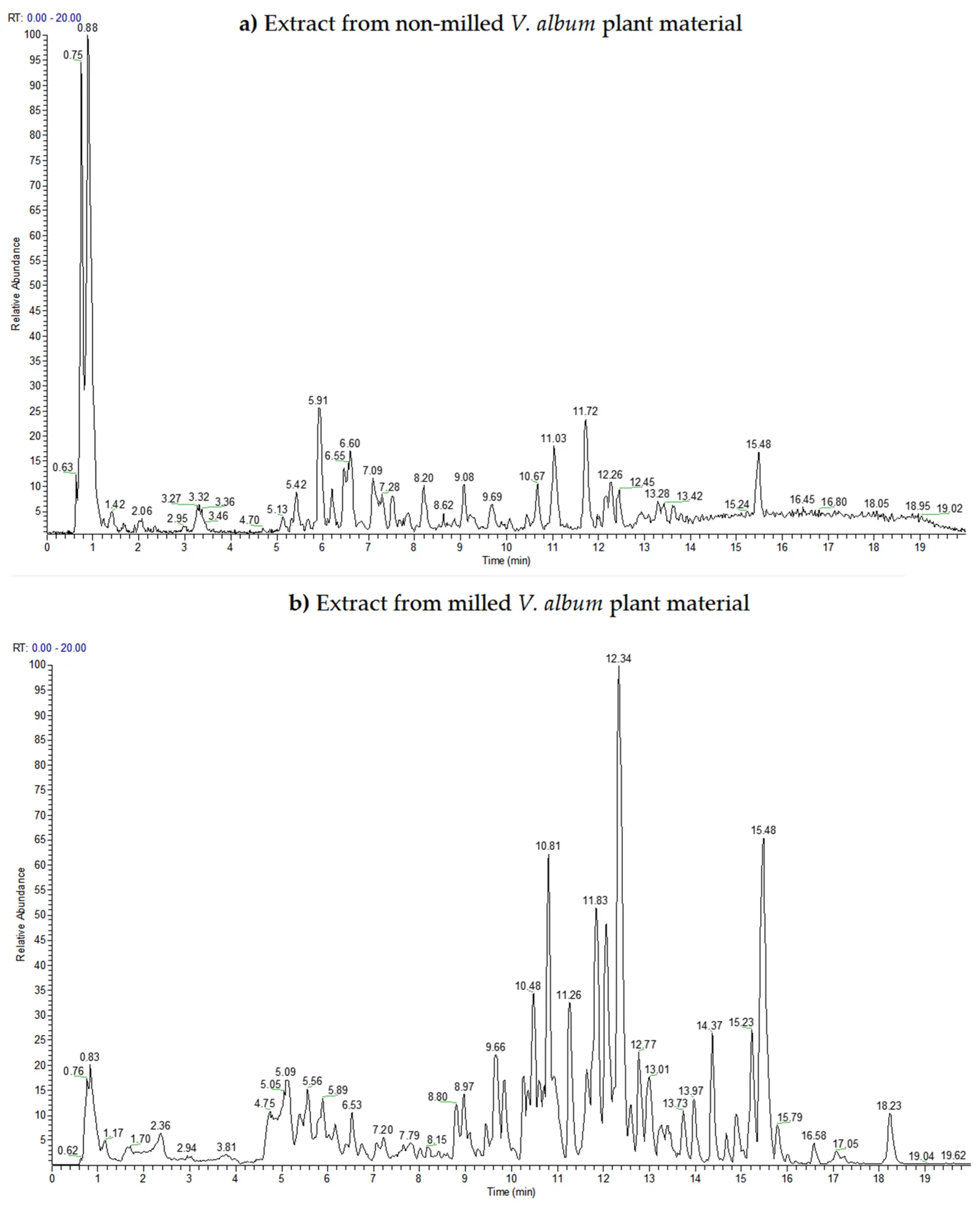
Representative ultra-high-performance liquid chromatography–mass spectrometry (UHPLC-MS) base-peak chromatograms of extracts from non-milled (**a**) and milled (**b**) *V. album* plant material.

A previously described UHPLC-DAD-MS/MS procedure [22,23,24] was applied with minor modifications. Chromatographic separation was performed on a Hypersil Gold C18 column (50 × 2.1 mm, 1.9 µm) at 25 °C using a Dionex Ultimate 3000 UHPLC+ system equipped with a diode-array detector (DAD) and an LCQ Fleet ion-trap mass spectrometer (Thermo Fisher Scientific, Waltham, MA, USA). The mobile phase consisted of (A) water containing 0.1% (*v*/*v*) formic acid and (B) methanol, at a flow rate of 0.250 mL min^−1^. The gradient was: 0–2 min, 10–30% B; 2–4 min, 30–35% B; 4–5 min, 35–40% B; 5–8 min, 40–60% B; 8–11 min, 60–90% B; 11–15 min, 90% B; 15–15.01 min, 90–10% B; and 15.01–20 min, 10% B. UV–Vis absorption spectra were recorded with the DAD over 200–800 nm. The UHPLC-DAD chromatograms shown in Figure 1 and Figure 2 were recorded at 280 and 330 nm, respectively, while Figure 3 presents the UHPLC-MS base-peak chromatograms.

Mass spectrometric analysis was performed using a three-dimensional ion trap with electrospray ionization (ESI) in negative-ion mode. The ESI source parameters were: source voltage, 4.5 kV; capillary voltage, −41 V; tube-lens voltage, −95 V; capillary temperature, 350 °C; and nitrogen sheath- and auxiliary-gas flows, 32 and 8 arbitrary units, respectively. Full-scan mass spectra were acquired over *m*/*z* 100–900. Tandem mass spectrometry was performed by data-dependent collision-induced dissociation (CID) of detected deprotonated molecular ions ([M–H]^–^), with normalized collision energy set at 30%. Before analysis, samples were filtered through a 0.45 µm membrane filter. The injection volume was 1 µL.

Xcalibur software (version 2.1) controlled the instrument and supported data acquisition and analysis. The assignation of the detected compounds was based on their retention times, DAD UV–Vis spectra, deprotonated molecular ions ([M–H]^–^), characteristic MS/MS fragmentation, comparison with used reference standards (apigenin, caffeic acid, chlorogenic acid, citric acid, hyperoside, isoquercitrin, kaempferol, luteolin, malic acid, neochlorogenic acid, quercetin, rosmarinic acid, rutin and tiliroside), data from available MS database (MassBank) and published spectral data regarding the metabolomic studies for different *Viscum album* extracts. The fragmentation details for compounds used as standards are provided in the Supplemental Material (Table S1).

Compounds not confirmed using authentic standards are therefore reported as tentative identifications. For compound annotation, the highest confidence was assigned to compounds matched with authentic standards. Assignments without authentic standards were considered tentative and were supported by the available UV–Vis, precursor-ion and MS/MS fragmentation data, together with comparison to published studies of *Viscum album* extracts.

The UHPLC-DAD-MS/MS analysis was used for qualitative comparative profiling and compound assignment. It was not designed or validated for quantitative determination of individual metabolites; consequently, chromatographic signal differences are not interpreted as quantitative extraction efficiencies for specific compounds.

### 2.6. Antimicrobial Tests

Antimicrobial activity was assessed by agar disc diffusion and by determining the minimum inhibitory concentration (MIC) in a broth microdilution assay. The test microorganisms comprised the Gram-positive bacteria *Bacillus cereus* ATCC 11778, *Bacillus subtilis* ATCC 6633, and *Staphylococcus aureus* ATCC 25923; the Gram-negative bacteria *Escherichia coli* ATCC 25922, *Klebsiella pneumoniae* ATCC 700603, *Proteus vulgaris* ATCC 8427, and *Pseudomonas aeruginosa* ATCC 27853; and the yeast *Candida albicans* ATCC 2091. Microorganisms were obtained from the Microbiology Laboratory, Faculty of Technology, Leskovac. Fresh cultures were used to prepare the inocula. Nutrient agar (Torlak, Belgrade, Serbia) was used for bacterial cultivation, whereas Sabouraud maltose agar (Torlak, Belgrade, Serbia) was used for *C. albicans*. Inoculum turbidity was adjusted to a 0.5 McFarland standard, corresponding to approximately 1–2 × 10^8^ CFU/mL for bacteria and 1–2 × 10^6^ CFU/mL for *C. albicans*.

For the disc-diffusion assay, sterile paper discs (5 mm diameter) were impregnated with 50 µL of each *V. album* extract and placed on agar plates previously inoculated with standardized microbial suspensions. The same stock concentration (100 mg/mL) was used for both extracts in the disc-diffusion assay, corresponding to 5 mg of dried extract per disc. Bacterial plates were incubated at 37 °C for 24 h, whereas *C. albicans* plates were incubated at 25 °C for 72 h. Antimicrobial activity was assessed by measuring inhibition-zone diameters, expressed in millimeters (mm) as mean ± standard deviation. Ceftriaxone (30 µg/disc), cefoxitin (30 µg/disc), ciprofloxacin (5 µg/disc), gentamicin (10 µg/disc), imipenem (10 µg/disc), and nystatin (100 U/disc) commercial discs were used as positive controls, as appropriate for the tested microorganisms. Pure DMSO was used as the negative solvent control.

MIC was determined by broth microdilution in sterile 96-well microtiter plates. Stock solutions were prepared by dissolving 100 mg of dried extract in 1 mL of DMSO, corresponding to a concentration of 100 mg/mL. MIC values expressed in µL/mL refer to the volume of the prepared stock solution per mL of assay medium. Accordingly, 1 µL/mL corresponds to 0.1 mg/mL of dried extract. Two-fold serial dilutions of each extract were prepared in Mueller–Hinton broth (Torlak, Belgrade, Serbia) for bacterial assays and Sabouraud maltose broth (Torlak, Belgrade, Serbia) for *C. albicans*, to give final extract concentrations of 0.25–128 µL/mL. A 100 µL aliquot of each serially diluted extract was combined with 100 µL of standardized microbial inoculum in each well, giving a final volume of 200 µL. For bacterial assays, the final inoculum was approximately 5 × 10^5^ CFU/mL. For *C. albicans*, the standardized inoculum before addition to the wells was approximately 1–2 × 10^6^ CFU/mL. Growth-control wells containing inoculum without extract and sterility-control wells containing broth without inoculum were included; DMSO served as the solvent control. Bacterial microtiter plates were incubated at 37 °C for 24 h, whereas *C. albicans* plates were incubated at 25 °C for 72 h. Microbial growth was then assessed by optical-density measurement using an EZ Read 400 microplate reader (Biochrom, Cambridge, UK). MIC was defined as the lowest extract concentration producing complete inhibition of microbial growth.

Antimicrobial experiments were performed with extracts from three independent extractions, and each independently obtained extract was tested in triplicate; the independently obtained extract was treated as the experimental/statistical unit. Results are reported as mean ± standard deviation.

### 2.7. Techno-Economic Analysis, Evaluation and Comparison of the MAE Techniques

All indicators for the MAE techniques were calculated according to the calculation framework of Nikolić et al. [22]. The analysis was conducted at a laboratory scale for batch processes.

Total electricity consumption per batch (in kWh) was calculated by the following equation:

*Total electricity consumption per batch* = *E*_MAE_ + *E*_Mill_ = *P*_MAE_ · *t*_MAE_ + *P*_Mill_ · *t*_Mill_ (3)

where *E*_MAE_ and *E*_Mill_ (in kWh) and *P*_MAE_ and *P*_Mill_ (in kW) indicate the electricity consumption values per batch and the electric power values for the laboratory microwave extraction unit and the laboratory mill, respectively; *t*_MAE_ and *t*_Mill_ (in h) are time of MAE and duration of milling, respectively.

Thermal conductance (in kW/K) was calculated as

*Thermal conductance* = *P*/*T* (4)

where *P* is the applied microwave power (in kW), and *T* is the absolute extraction temperature (in K).

Heat capacity (in kWh/K) was calculated by the following equation:

*Heat capacity* = *Total electricity consumption per batch*/*T* (5)

Specific heat capacity (in kWh/K per kg solvent per kg plant material) was calculated according to the following equation:

*Specific heat capacity* = *Heat capacity*/*m_liq_* (6)

where *m_liq_* is the mass of the liquid extraction medium per mass of plant material (kg solvent per kg plant material). For 1000 mL of 70% (*v*/*v*) ethanol, the solvent-system mass used in the calculation was 0.853 kg (8.53 g solvent per g of plant material for a 100 g batch).

The mass of CO_2_ generated during the total process, afterwards released into the atmosphere, was calculated as

*m* (CO_2_) = 800 · *Total electricity consumption per batch* (7)

Total energy input (in kJ) is calculated as

*Total energy input* = *Energy input*_MAE_ + *Energy input*_Mill_ (8)

where *Energy input*_MAE_ and *Energy input*_Mill_ are energy input values for the MAE and milling procedures, respectively.

Specific energy consumption per kg dry matter (in kJ/kg d.m.) was calculated by the following equation:

*Specific energy consumption per kg dry matter* = *Total energy input* · *m (dry plant material)* (9)

Specific energy consumption per kg TEM (in kJ/kg TEM) was calculated by the following equation:

*Specific energy consumption per kg TEM* = *Total energy input* · *TEM extracted per batch* (10)

The costs of the proposed processes are calculated according to the following equations:

*Electricity cost per batch* = *Total electricity consumption per batch* · *Average electricity price* (11)

*Electricity cost per kg TEM* = *Electricity cost per batch*/*TEM extracted per batch* (12)

### 2.8. Statistical Analysis

SPSS 23.0 (IBM, Armonk, NY, USA) was used for statistical analysis. Normality was assessed using the Shapiro–Wilk test, and results are presented as mean ± standard deviation. Because the antimicrobial data were not normally distributed, non-parametric comparisons were performed using the Kruskal–Wallis H test. For each extract, differences among the eight tested microorganisms were evaluated separately; when an overall difference was significant, post hoc comparisons were performed using Dunn’s method with Bonferroni correction. Data for both extracts were also compared separately for each microorganism using the Kruskal–Wallis H test. Differences were considered statistically significant at *p* ≤ 0.05.

## 3. Results and Discussion

### 3.1. Total Extractive Matter (TEM)

Table 1 presents TEM values and their percentage ratios relative to the highest overall TEM values observed across both plant-material preparations.

As shown in Table 1, TEM value increased with extraction time and reached a plateau at 20 min, with no further increase at 30 min. Under identical MAE conditions, the milled sample showed a higher TEM value than the non-milled sample, indicating a beneficial effect of milling on extraction performance. Based on the observed plateau, 20 min was selected for the subsequent comparison of the two preparations.

### 3.2. Qualitative UHPLC-DAD-MS/MS Phytochemical Profiling

Figure 1, Figure 2 and Figure 3 show representative UHPLC chromatograms of both extracts. Table 2 summarizes the compounds detected under the applied analytical conditions together with their retention times, UV–Vis and MS/MS characteristics, and proposed assignments. The comparison is qualitative; peak presence/absence and compound assignments are used to describe compositional profiles and are not used to quantify the extraction efficiency of individual metabolites.

Compounds confirmed using authentic standards are indicated in Table 2. Other compounds were tentatively assigned using retention times, UV spectra, MS/MS fragmentation patterns, and comparison with published data. The following literature-based fragmentation rules were used to support tentative assignments. Accordingly, the discussion distinguishes standard-confirmed compounds from tentative literature-supported assignments. Where authentic standards were unavailable, the proposed identities are interpreted cautiously, and the available analytical evidence does not permit definitive discrimination between all possible structural isomers.

According to the fragmentation rules in the literature for flavonoids, the most common neutral losses characteristic in negative ion mode ESI-MS/MS analysis are 15 Da (CH_3_·), 16 Da (CH_4_), 18 Da (H_2_O), 28 Da (CO), 42 Da (C_2_H_2_O), 44 Da (CO_2_), 46 Da (H_2_O + CO), 56 Da (2 CO), 62 Da (CO_2_ + H_2_O), 74 Da (H_2_O + 2 CO) and 84 Da (3 CO) [25]. For monomethoxylated flavonoid aglycones, the most prevalent ions are [M–H–CH_3_]^–^ (Δ 15 Da), [M–H–CH_3_–CO]^–^ (Δ 43 Da = 15 + 28) and [M–H–CH_3_–HCO]^–^ (Δ 44 Da = 15 + 29), while for the polymethoxylated flavonoid aglycones, the most prevalent ions are [M–H–CH_3_]^–^ (Δ 15 Da), [M–H–2CH_3_]^–^ (Δ 30 Da) and [M–H–2CH_3_–CO]^–^ (Δ 58 Da = 30 + 28) [26]. The sugar moieties from flavonoid O-glycosides, such as hexoses, deoxy-hexose, pentoses, and uronic acid residues, were identified by the neutral losses of 162 Da [C_6_H_10_O_5_], 146 Da [C_6_H_10_O_4_], 132 Da [C_5_H_8_O_4_], and 176 Da [C_6_H_8_O_6_], respectively [13]. The presence of 3-hydroxy-3-methylglutaroyl (HMG) esters in flavonoids was characterized by neutral losses of 62 Da [M–H–CO_2_–H_2_O]^–^, 102 Da [M–H–C_4_H_6_O_3_]^–^ and 144 Da [M–H–HMG]^–^ [27]. Fragments at *m*/*z* 299 and *m*/*z* 209 are characteristic of the hydroxy-dimethoxyflavanone aglycone and sinapylalcohol moieties, respectively [27].

The main compound classes in both extracts were organic acids, phenolic acids, and flavonoid glycosides, followed by chlorophyll derivatives, coumarin glycosides, and sesquiterpenoids.

**Table 2:** Ultra-high-performance liquid chromatography with diode-array detection and tandem mass spectrometry (UHPLC-DAD-MS/MS) characteristics and assignments of compounds detected in microwave-assisted extraction (MAE) extracts from non-milled (A) and milled (B) *V. album* plant material.

Peak No.	*t*_R_, min	λ_max_, nm	[M–H]^–^, *m*/*z*	MS/MS Fragment Ions	A	B	Assignment	Reference
1	0.75	-	191	173,129,127,111,85 (100)	+	+	Quinic acid	[23,28]
2	0.83	-	133	115 (100%),87	+	+	Malic acid	Standard; [23]
3	0.92	-	191	173,111 (100%),85	+	+	Citric acid	Standard; [23,28]
4	1.13	-	161	143,101,99 (100%),57	+	+	3-Hydroxy-3-methylglutaric acid	[29]
5	2.04	-	161	143 (100%),115,71	+	+	3- or 4-Hydroxy-2-oxoglutaric acid	[30]
6	2.33	297sh 325	353	191 (100%),179,173,135	-	+	Neochlorogenic acid	Standard
7	3.32	-	339	177 (100%)	+	-	Esculetin glucoside/galactoside	[31]
8	3.83	258	351	286,249,207,143,125 (100%)	-	+	Hydroxyflavanone HMG derivative	
9	3.94	304sh 313	337	163 (100%),119	+	+	3-*O*-*p*-coumaroyl-quinic acid	[27,32]
10	5.13	300sh 326	353	191 (100%)	+	+	Chlorogenic acid	Standard
11	5.60	301sh 322	353	191,179,173 (100%),135	-	+	Cryptochlorogenic acid	[27,33]
12	5.94	222 267	549	503 (100%)	+	+	Sinapylalcohol pentosyl-hexoside formic acid adduct	[27]
13	6.44	289sh 315	337	191,173 (100%),163,111	+	+	4-*O*-*p*-coumaroyl-quinic acid	[27,32]
14	6.55	-	563	517 (100%)	+	+	Sinapylalcohol deoxyhexosyl/rhamnosyl-hexoside formic acid adduct	[27]
15	6.60	232 310	431	385,223,179 (100%),161,153	+	+	Roseoside formate adduct	[34,35,36]
16	6.74	-	367	193,191 (100%),173	+	+	5-*O*-feruloyl-quinic acid	[27,33]
17	7.10	-	627	581 (100%),453	+	+	Succinoyl-caffeoylquinic acid derivative	[27]
18	8.20	279 340	509	463(100%)	+	+	Quercetin-hexoside formate adduct	[22,27,31,37]
19	8.80	284	753	591,489,447,285 (100%)	-	+	Tetrahydroxyflavone-dihexoside-HMG derivative	[23,24,37]
20	8.97	286	753	591,489,447,285 (100%)	-	+	Tetrahydroxyflavone-dihexoside-HMG derivative	[23,24,37]
21	9.10	295	547	311 (100%)	+	-	Hydroxybenzoylquinic acid derivative	[27]
22	9.65	-	639	593 (100%)	+	-	4′-Hydroxy-5,7-dimethoxyflavanone-pentosyl-hexoside formate adduct or kaempferol-3-*O*-rutinoside formate adduct	[27,37,38]
23	9.70	234 291	625	579,399 (100%)	-	+	Sakuranetin-pentosyl-hexoside derivative	[27]
24	9.84	254 355	753	691,651,609 (100%)	-	+	Tetrahydroxy-methoxyflavone-HMG-pentosyl-hexoside	[27,37]
25	10.45	254 357	315	279,163,149 (100%),107	+	+	Rhamnetin or isorhamnetin	[39]; MSBNK-RIKEN_ReSpect-PS041007; CID:5281691
26	10.66	240 340	427	381 (100%),366	+	-	n.i.	
27	10.81	234 288	737	675,635,593 (100%),299	-	+	4′-Hydroxy-5,7-dimethoxyflavanone-HMG-pentosyl-hexoside	[27,37]
28	10.97	240 269 304	605	299 (100%)	-	+	4′-Hydroxy-5,7-dimethoxyflavanone-acetylpentosyl-pentose	[37]
29	11.03	326	689	643 (100%)	+	-	n.i.	
30	11.63	-	623	491,473,461,383,328/329 (100%),313,299/301,271	+	+	Rhamnazin-pentosyl-hexoside	[27,37,39]
31	11.71	256 357	737	623 (100%)	+	+	Trihydroxy-dimethoxyflavone-pentosyl-hexoside derivative	[27,37]
32	11.84	-	723	579 (100%)	-	+	Tetrahydroxyflavone-HMG-pentosyl-hexoside (kaempferol or sakuranetin derivative)	[27]
33	12.07	-	723	661,621,579 (100%),285	-	+	Sakuranetin-HMG-pentosyl-hexoside	[37]
34	12.18	291	591	447,285 (100%)	+	+	Sakuranetin-HMG-hexoside or kaempferol-HMG-hexoside	[27,37]
35	12.32	357	867	723,661,621,579 (100%),561,285	-	+	Sakuranetin-di-HMG-pentosylhexoside	[27]
36	12.45	257 356	329	314 (100%),271	+	+	Rhamnazin	[37,39]
37	12.59	361	737	593 (100%),299,285	-	+	4′-Hydroxy-5,7-dimethoxyflavanone-HMG-pentosyl-hexoside	[27,37]
38	12.59	362	507	461,299 (100%)	+	+	4′-Hydroxy-5,7-dimethoxyflavanone-hexoside formic acid adduct	[37]
39	12.77	360	737	593 (100%),299	-	+	4′-Hydroxy-5,7-dimethoxyflavanone-HMG-pentosyl-hexoside	[27,37]
40	12.96	359	605	561,461,299 (100%)	-	+	4′-Hydroxy-5,7-dimethoxyflavanone-HMG-hexoside	[27]
41	13.26	-	329	314 (100%),291,229,211,171	+	+	Trihydroxy-dimethoxyflavone	[31,37]
42	13.73	370	299	119 (100%)	-	+	4′-Hydroxy-5,7-dimethoxyflavanone	[37]; MB: BS003739 MSBNK-BS-BS003739; PubChem CID 5271551
43	13.97	278	343	299 (100%)	-	+	Dihydroxy-trimethoxyflavone	[37]
44	15.45	280	610	564 (100%)	+	+	n.i.	
45	17.70	400 654	-	-	-	+	Unidentified chlorophyll derivative	[40]
46	17.80	410 665	-	-	-	+	Unidentified chlorophyll derivative	[40]
47	18.00	418 654	-	-	-	+	Unidentified chlorophyll derivative	[40]
48	18.24	-	535	466,451,397,383,315 (100%)/313	-	+	Tentative: tetrahydroxy-methoxyflavone derivative	

Abbreviations: A, non-milled plant material; B, milled plant material; sh, shoulder; n.i., not identified; MB, MassBank record. Compounds not confirmed by comparison with authentic standards are reported as tentative assignments based on retention behavior, UV–Vis spectra, MS/MS fragmentation, and literature data.

The presence of organic acids is noticeable. Peak No. 2 (both samples) at *t*_R_ = 0.83 min was identified as malic acid by comparison with an authentic standard and according to the [M–H]^–^ ion at *m*/*z* 133, which generated fragment ions at *m*/*z* 115 and 87 [23]. Peak No. 3 (both samples) at *t*_R_ = 0.92 min was identified as citric acid by comparison with an authentic standard. The [M–H]^–^ ion at *m*/*z* 191 generated fragment ions at *m*/*z* 173, 111, and 85 [23,28,36,41]. Peak No. 4 (both samples) at *t*_R_ = 1.13 min showed a [M–H]^–^ ion at *m*/*z* 161 and fragment ions at *m*/*z* 143, 101, 99, and 57. This fragmentation was consistent with 3-hydroxy-3-methylglutaric acid [29]. Similarly, peak No. 5 (both samples) at *t*_R_ = 2.04 min has [M–H]^–^ ion at *m*/*z* 161 provided fragment ions at *m*/*z* 143 [M–H–(18)–H_2_O]^–^, *m*/*z* 115 [M–H–(46)–H_2_O–CO]^–^, and *m*/*z* 71 [M–H–(90)–H_2_O–CO–CO_2_]^–^, so it was tentatively assigned as either 3-hydroxy-2-oxoglutaric acid or 4-hydroxy-2-oxoglutaric acid [30].

Quinic acid and its derivatives are also present in the extracts. Peak No. 1 (both samples) at *t*_R_ = 0.75 min showed a [M–H]^–^ ion at *m*/*z* 191 with fragment ions at *m*/*z* 173, 129, 127, 111, and 85, so it was assigned as quinic acid [23,28,36,41]. This acid and its derivatives are commonly found in *V. album* extracts [37]. For instance, the compounds No. 6, No. 10 and No. 11 are three caffeoylquinic acid isomers. Peak No. 6 (sample B) at *t*_R_ = 2.60 min was identified as neochlorogenic (3-*O*-caffeoylquinic) acid by comparison with an authentic standard. Its [M–H]^–^ ion at *m*/*z* 353 generated fragment ions at *m*/*z* 191, 179, 173, and 135. Peak No. 10 (both samples) at *t*_R_ = 5.13 min was identified as 5-*O*-caffeoylquinic (chlorogenic) acid by comparison with an authentic standard. The [M–H]^–^ ion at *m*/*z* 353 generated the characteristic fragment ion at *m*/*z* 191. Peak No. 11 (sample B) at *t*_R_ = 5.60 min showed a [M–H]^–^ ion at *m*/*z* 353 and fragment ions at *m*/*z* 191, 179, 173, and 135. This fragmentation pattern is consistent with cryptochlorogenic (4-*O*-caffeoylquinic) acid [27,33]. Neochlorogenic, chlorogenic and cryptochlorogenic acids are previously described in *V. album* extracts [27,37]. Compounds No. 9 and No. 13 are two *p*-coumaroylquinic acid isomers. Peak No. 9 (both samples) at *t*_R_ = 3.94 min showed a [M–H]^–^ ion at *m*/*z* 337 and fragments at *m*/*z* 163 and 119, so it is assigned as 3-*O*-*p*-coumaroylquinic acid [27,32]. Peak No. 13 (both samples) at *t*_R_ = 6.44 min showed a [M–H]^–^ ion at *m*/*z* 337 with fragment ions at *m*/*z* 191, 173, 163, and 111, characteristic of 4-*O*-*p*-coumaroylquinic acid [32]. Additionally, peak No. 16 (both samples) at *t*_R_ = 6.74 min showed a [M–H]^–^ ion at *m*/*z* 367 and fragment ions at *m*/*z* 193, 191, and 173. These ions are consistent with ferulic acid- and quinic acid-related fragments, so this compound was assigned as 5-*O*-feruloylquinic acid [27,33]. These acids have also been previously detected in *V. album* extracts [27]. Peak No. 17 (both samples) at *t*_R_ = 7.10 min showed a [M–H]^–^ ion at *m*/*z* 627 and fragment ions at *m*/*z* 581 and 453. According to the literature, this compound is a succinoyl-caffeoylquinic acid derivative, found in *V. album* extracts [27]. Peak No. 21 (sample A) at *t*_R_ = 9.10 min showed a [M–H]^–^ ion at *m*/*z* 547 and a dominant fragment at *m*/*z* 311. Because *m*/*z* 311 has been reported for hydroxybenzoylquinic acid isomers, this compound is classified as a hydroxybenzoylquinic acid derivative, also found in *V. album* and its extracts [27].

Compounds No. 12 and No. 14 are two sinapyl alcohol glycosides common in *V. album* [27]. Peak No. 12 (both samples) at *t*_R_ = 5.94 min showed a [M–H]^–^ ion at *m*/*z* 549 and a fragment at *m*/*z* 503 after a neutral loss of 46 Da. Because *m*/*z* 503 and its reported fragmentation are consistent with sinapyl alcohol pentosyl-hexoside, this compound is assigned as a sinapyl alcohol pentosyl-hexoside formate adduct [27]. Moreover, peak No. 14 (both samples) at *t*_R_ = 6.55 min showed a [M–H]^–^ ion at *m*/*z* 563 and a fragment at *m*/*z* 517 after a 46 Da neutral loss. Its fragmentation resembled that of compound 12, with a 14 Da difference in the precursor ion. Therefore, this compound 14 was assigned as a sinapyl alcohol deoxyhexosyl/rhamnosyl-hexoside formate adduct [27].

Peaks No. 19 and 20 (sample B), at *t*_R_ = 8.80 and 8.97 min, were isomers with [M–H]^–^ ions at *m*/*z* 753 and fragments at *m*/*z* 591, 489, 447, and 285. The sequential losses were consistent with hexosyl and HMG moieties, while *m*/*z* 285 indicated a tetrahydroxyflavone aglycone [23,24,27,37]. Therefore, these compounds are assigned as tetrahydroxyflavone-dihexoside-HMG derivatives, previously found in *V. album* extracts [27]. Similarly, peak No. 24 (sample B) at *t_R_* = 9.84 min showed a [M–H]^–^ ion at *m*/*z* 753 and fragments at *m*/*z* 691, 651, and 609, consistent with the HMG-containing flavonoid derivative. This compound is assigned as tetrahydroxy-methoxyflavone-HMG-pentosyl-hexoside, a metabolite common in *V. album* and its extracts [27,37].

Compounds No. 23, No. 33, and No. 35 represent sakuranetin-glycoside derivatives. Peak No. 23 (sample B) at *t_R_* = 9.70 min showed a [M–H]^–^ ion at *m*/*z* 625 and fragments at *m*/*z* 579 and 399, representing a sakuranetin-pentosyl-hexoside derivative [27,37], while peak No. 33 (sample B) at *t_R_* = 12.07 min with a [M–H]^–^ ion at *m*/*z* 723 and fragments at *m*/*z* 661, 621, 579, and 285 indicate that this compound is sakuranetin-HMG-pentosyl-hexoside [37]. Peak No. 35 (sample B) at *t_R_* = 12.32 min with the [M–H]– ion at *m*/*z* 867 has a fragmentation pattern similar to compound 33, so it is assigned as a sakuranetin-di-HMG-pentosyl-hexoside [27]. These sakuranetin derivatives are known V. album metabolites and are present in its extracts [27,37]. These sakuranetin derivatives are known *V. album* metabolites and are present in its extracts [27,37].

Compounds No. 32 and No. 34 are tetrahydroxyflavone-glycoside derivatives. Peak No. 32 (sample B) at *t*_R_ = 11.84 min showed a [M–H]^–^ ion at *m*/*z* 723 and a fragment at *m*/*z* 579 after a 144 Da neutral loss consistent with an HMG moiety. Based on the literature data, this compound is either kaempferol-HMG-pentosyl-hexoside or sakuranetin-HMG-pentosyl-hexoside, both present in *V. album* extracts [27]. Consequently, peak No. 34 (both samples) at *t*_R_ = 12.18 min with a [M–H]^–^ ion at *m*/*z* 591 and fragments at *m*/*z* 447 and 285 can represent sakuranetin-HMG-hexoside or kaempferol-HMG-hexoside, both known *V. album* metabolites [27,37].

By looking at the overall table, compounds No. 22, No. 27, No. 28, No. 37, No. 38, No. 39 and No. 40 are derived from compound 42. Peak No. 42 (sample B) at *t_R_* = 13.73 min with a [M–H]^–^ ion at *m*/*z* 299 and a fragment at *m*/*z* 119 represents 4′-hydroxy-5,7-dimethoxyflavanone [MSBNK-BS-BS003739; PubChem CID 5271551], a common metabolite of *V. album* whose derivatives are present in its extracts [27,37]. Peaks No. 37 and 39 (sample B), at *t*_R_ = 12.59 and 12.77 min, are isomers with a [M–H]^–^ ion at *m*/*z* 737 and fragments at *m*/*z* 593 and 299, confirming these compounds are two 4′-hydroxy-5,7-dimethoxyflavanone-HMG-pentosyl-hexosides [27,37]. Additionally, peak No. 27 (sample B) at *t_R_* = 10.81 min with a [M–H]^–^ ion at *m*/*z* 737 and fragments at *m*/*z* 675, 635, 593, and 299 confirms that this compound is 4′-hydroxy-5,7-dimethoxyflavanone-HMG-pentosyl-hexoside [27,37]. Peak No. 38 (both samples) at *t*_R_ = 12.59 min showed an ion at *m*/*z* 507, interpreted as a formate adduct, with fragments at *m*/*z* 461 and 299, confirming that this compound is a 4′-hydroxy-5,7-dimethoxyflavanone-hexoside formate adduct [37]. Peak No. 40 (sample B) at *t*_R_ = 12.96 min showed a [M–H]^–^ ion at *m*/*z* 605 and fragments at *m*/*z* 561, 461, and 299, corresponding to 4′-hydroxy-5,7-dimethoxyflavanone-HMG-hexoside [27]. Peak No. 28 (sample B) at *t*_R_ = 10.97 min has a [M–H]^–^ ion at *m*/*z* 605, producing a dominant fragment ion at *m*/*z* 299. The neutral loss of 306 Da is consistent with releasing one acetyl group (42 Da) and two pentosyl residues (2 × 132 Da). Therefore, this compound is assigned as 4′-hydroxy-5,7-dimethoxyflavanone-acetylpentosyl-pentose [37].

Peak No. 22 (sample A) at *t*_R_ = 9.65 min showed a [M–H]^–^ ion at *m*/*z* 639 and a fragment at *m*/*z* 593 after a 46 Da neutral loss, suggesting it is a formate adduct. Since the fragment at *m*/*z* 593 indicates 4′-hydroxy-5,7-dimethoxyflavanone-pentosyl-hexoside [27,37], this compound is presumed to be its formate adduct. However, the fragment at *m*/*z* 593 can also indicate kaempferol-3-*O*-rutinoside [27], so this compound can also represent its formate adduct. Both compounds are found in *V. album* extracts [27,37].

Peak No. 43 (sample B) at *t*_R_ = 13.97 min showed a [M–H]^–^ ion at *m*/*z* 343 and a fragment at *m*/*z* 299. Since the *m*/*z* 299 indicates hydroxy-dimethoxyflavanone aglycone, this compound can be assigned as dihydroxy-trimethoxyflavone, also found in *V. album* extracts [37].

Peak No. 18 (both samples) at *t*_R_ = 8.20 min showed a [M–H]^–^ ion at *m*/*z* 509 and a fragment at *m*/*z* 463 after a 46 Da neutral loss. Because *m*/*z* 463 is characteristic of quercetin hexosides, compound 18 was tentatively assigned as a quercetin-hexoside formate adduct [22,31,37,42,43,44]. This compound is also common in *V. album* extracts [37].

Compounds No. 36 and No. 41 are isomers present in both samples, with the same [M–H]^–^ ion at *m*/*z* 329 and the most pronounced fragment ions at *m*/*z* 314, suggesting loss of 15 Da due to release of a CH_3_ group. However, compound No. 36 also has a generated ion at *m*/*z* 271, suggesting that the ion at *m*/*z* 314 loses 43 Da due to the release of a CH_3_–CO moiety. This fragmentation pattern suggests that this compound is rhamnazin, frequently detected in *V. album* extracts [39]. Compound No. 41 has a different fragmentation pattern, providing ions at *m*/*z* 314, 229, and 211. The data support a trihydroxy-dimethoxyflavone assignment, but they do not distinguish positional isomers; compound 41 was therefore retained at the class level [31,37]. Peak No. 30 (both samples) at *t*_R_ = 11.63 min showed a [M–H]^–^ ion at *m*/*z* 623 and fragment ions including *m*/*z* 491, 473, 461, 329/328, 313, 301/299, and 271. The losses of pentosyl and hexosyl moieties and the aglycone-related ion at *m*/*z* 329 support a trihydroxy-dimethoxyflavone glycoside. Since rhamnazin is already detected and has a [M–H]^–^ ion at *m*/*z* 329 and characteristic fragment ions at *m*/*z* 313 and *m*/*z* 271, this compound is assigned as rhamnazin-pentosyl-hexoside, which is detected in different *V. album* extracts [27,37]. Consequently, peak No. 31 (both samples) at *t*_R_ = 11.71 min with a [M–H]^–^ ion at *m*/*z* 737 and a fragment at *m*/*z* 623 is classified as a trihydroxy-dimethoxyflavone-pentosyl-hexoside derivative [27,37].

Peak No. 25 (both samples) at *t*_R_ = 10.45 min occurs at λ_max_ = 254 and 357 nm, suggesting a flavonoid-type compound. It has [M–H]^–^ ion at *m*/*z* 315 and fragment ions at *m*/*z* 279 [M–H–(36)]^–^, i.e., [M–H–(2 H_2_O)]^–^, *m*/*z* 163 [M–H-(152) = M–H–(36)–(116) = M–H–(36)–(84)–(32)]^–^, i.e., [M–H–(2 H_2_O)–(3 CO)–(CH_3_OH)]^–^, *m*/*z* 149 [M–H–(166) = M–H–(36)–(116)–(14) = M–H–(36)–(84)–(32)–(14)]^–^, i.e., [M–H–(2 H_2_O)–(3 CO)–(CH_3_OH)–(CH_2_)]^–^, and *m*/*z* 107 [M–H–(208) = M–H–(36)–(116)–(14)–(42)] = M–H–(36)–(84)–(32)–(14)–(42)]^–^, i.e., [M–H–(2 H_2_O)–(3 CO)–(CH_3_OH)–(CH_2_)–(C_2_H_2_O)]^–^. These data support a tetrahydroxy-methoxyflavone assignment. The MS/MS data for this compound were interpreted conservatively because closely related methoxylated flavonoid isomers can show overlapping neutral-loss and ring-cleavage patterns in negative-ion mode [25,26,44]. Therefore, this compound can be tentatively classified as rhamnetin or isorhamnetin; both flavonoids are present in *V. album* products [39].

Peak No. 8 (sample B) at *t*_R_ = 3.83 min showed a [M–H]^–^ ion at *m*/*z* 351 and fragment ions at *m*/*z* 286, 249, 207, 143, and 125. The losses observed at *m*/*z* 249 and 207 are compatible with fragmentation reported for HMG-containing flavonoid derivatives, while the ion at *m*/*z* 125 has also been described in negative-ion spectra of hydroxyflavonoids [27,42]. Because the available MS/MS data do not support an unambiguous structure, compound 8 was conservatively classified as a hydroxyflavanone HMG derivative.

Peak No. 7 (sample A) at *t*_R_ = 3.32 min showed a [M–H]^–^ ion at *m*/*z* 339 and a fragment ion at *m*/*z* 177, corresponding to a neutral loss of 162 Da. Because *m*/*z* 177 is consistent with esculetin, compound 7 was tentatively assigned as an esculetin hexoside (glucoside or galactoside) [31].

Peak No. 15 (both samples) at *t*_R_ = 6.60 min showed an ion at *m*/*z* 431, interpreted as a formate adduct, and a fragment at *m*/*z* 385 after loss of 46 Da. Further fragmentation, including *m*/*z* 223, was consistent with roseoside/vomifoliol-related ions reported in the literature. Compound 15 was therefore tentatively assigned as a roseoside formate adduct [34,35,36].

Peak No. 48 (sample B) at *t*_R_ = 18.24 min showed a [M–H]^–^ ion at *m*/*z* 535 and fragment ions at *m*/*z* 466, 451, 397, 383, and 315/313. The lower-mass ions are consistent with fragmentation of methoxylated flavonoid-related structures reported in the literature. Because the available MS/MS data do not support an unambiguous complete structure, compound 48 was conservatively classified as a tetrahydroxy-methoxyflavone derivative [25,27].

Peaks No. 45–47 (sample B), at *t*_R_ = 17.70, 17.80, and 18.00 min, were classified as unidentified chlorophyll derivatives on the basis of their chromatographic and spectral characteristics [40]. Although these three chlorophyll-based derivatives were clearly observed in the UV–Vis chromatograms based on their Soret and Q bands, they remained uncharacterized by MS. The absence of informative MS signals under the applied analytical conditions may be related to limited ionization efficiency in negative electrospray mode (ESI−); however, the available data do not allow a definitive explanation. Therefore, these peaks are reported only as tentatively classified chlorophyll-related signals based on their UV–Vis characteristics, without assigning specific molecular structures.

The detected compounds belong to chemical classes associated with various biological activities. Quinic-acid derivatives, including *p*-coumaroylquinic acids, have been reported to possess antioxidant and antimicrobial properties, while chlorogenic-acid isomers have been associated with antioxidant, antimicrobial, anti-inflammatory, and other biological effects [23,45,46,47,48].

### 3.3. Antimicrobial Activity

Inhibition-zone results showed differences in susceptibility to the two *V. album* extracts (Table 3). Both extracts were tested at the same concentration (100 mg/mL), with 50 µL applied per disc (5 mg of dried extract per disc), ensuring a comparison based on equal nominal extract amounts. The extract obtained by MAE from milled plant material produced larger inhibition zones than the extract from non-milled material against all microorganisms for which inhibition was observed. The largest zones for the milled-material extract were recorded against *B. subtilis* and *B. cereus*, followed by *K. pneumoniae* and *S. aureus*. *P. aeruginosa* was the least susceptible microorganism to the milled-material extract, while no inhibition was observed with the non-milled-material extract. *C. albicans* was also more sensitive to the milled-material extracts. The inhibition zone obtained with the milled-material extract against *C. albicans* was larger than that recorded for nystatin under the specific assay conditions used. Since the Shapiro–Wilk test indicated that the values of inhibition zones were not normally distributed, the Kruskal–Wallis H test (one-way ANOVA on ranks) was performed. Significant differences in inhibition zone values were reported against all eight tested microorganisms for extract produced from milled (χ2 (7, *N* = 24) = 21.419, *p* = 0.003) and non-milled material (χ2 (7, *N* = 24) = 21.733, *p* = 0.003). In the case of extract produced from milled material, according to the post hoc comparisons using Dunn’s method with a Bonferroni correction for multiple tests, only the differences in inhibition zone values between *P. aeruginosa* and *B. cereus* (*p* = 0.042) and *P. aeruginosa* and *B. subtilis* (*p* = 0.01) were statistically significant. On the other hand, for the extract produced from the non-milled material, the post hoc test revealed significant differences only between *P. aeruginosa and B. subtilis* (*p* = 0.007).

The MIC results showed a similar trend (Table 4). The milled-material extract showed MIC values one two-fold dilution step below those of the non-milled-material extract against *B. cereus*, *B. subtilis*, *C. albicans*, *E. coli*, *K. pneumoniae* and *S. aureus*. The MICs were identical for both extracts against *P. aeruginosa* and *P. vulgaris*. *C. albicans* showed the lowest MIC values for both extracts. The Shapiro–Wilk test indicated the MIC values were not normally distributed. For all eight tested microorganisms, the Kruskal–Wallis test indicated that there was a significant difference in MIC values across both extracts obtained by MAE from the milled (χ^2^ (7, *N* = 24) = 21.226, *p* = 0.003) and non-milled plant material (χ^2^ (7, *N* = 24) = 19.252, *p* = 0.007). According to the *post hoc* comparisons using Dunn’s method with a Bonferroni correction for multiple tests, only the differences in MIC values obtained with the extract from the milled plant material for *C. albicans* and *P. aeruginosa* (*p* = 0.02) and *C. albicans* and *P. vulgaris* (*p* = 0.02) were deemed statistically significant. In the case of extract produced from non-milled material, no statistically significant differences were found. For *B. cereus*, *B. subtilis*, *C. albicans*, *E. coli*, *K. pneumoniae* and *S. aureus* the results of the Kruskal–Wallis test showed significant differences in MIC values (χ^2^ (1, *N* = 6) = 3.857, *p* = 0.05); yet, no significant differences in MIC values for *P. aeruginosa* (χ^2^ (1, *N* = 6) = 0, *p* = 1) and *P. vulgaris* (χ^2^ (1, *N* = 6) = 0, *p* = 1) were noticed.

According to the literature, the antimicrobial activity of *V. album* preparations depends strongly on plant part, host plant, geographical origin, extraction solvent and conditions, extract concentration, test microorganism, and assay design [1,49,50,51,52,53,54]. Nevertheless, the present results are consistent with the broader literature showing measurable antibacterial and anticandidal activity of *V. album* extracts and marked microorganism-dependent susceptibility.

Bilonozhko et al. [54] reported modest activity of aqueous *V. album* extracts against *S. aureus* and little or no activity against some Gram-negative bacteria, whereas Hussain et al. [51] found that antimicrobial effectiveness varied substantially with solvent and plant part. Shah et al. [1] likewise demonstrated that stem, leaf, and fruit extracts differed in their activity and that organic-solvent extracts were generally more active than aqueous preparations. Kusi et al. [52], Sengul et al. [49], Ertürk [50], and Oubaid et al. [53] further showed that susceptibility patterns differ considerably among the tested microbial species. In the present study, the lower MIC values for six microorganisms, together with the generally larger inhibition zones, describe the antimicrobial performance of the extract obtained after the defined milling pretreatment under the tested conditions. These biological results are treated independently from TEM yield and from the qualitative UHPLC-DAD-MS/MS profile.

The observed antimicrobial activity may reflect the combined contributions of multiple phytochemicals, including possible additive or synergistic interactions caused by these compounds; these effects remain hypothetical and require confirmation through targeted bioactivity and interaction studies.

However, these initial results suggest that further investigation of these MAE extracts as plant-derived antimicrobial preparations should be performed, with a greater focus on the use of milled plant material.

### 3.4. Techno-Economic Analysis, Evaluation and Comparison of the MAE Techniques

With the goal of scaling up the appropriate process on an industrial level, it is necessary to compare energy demand, environmental impact and cost efficiency of the proposed extraction methodologies.

Both extractions were performed at 500 W, 60 °C and 20 min. Since the amount of solvents was the same in both cases, the solvent cost was not included in the final financial calculation. The analysis was conducted at a laboratory scale for batch processes and focused on energy consumption and extraction efficiency; specific factors are listed in Table 5.

Since milling is the step that requires energy, the values regarding the total electricity consumption per batch, total energy input, specific energy consumption per kg of dry matter, heat capacity, specific heat capacity and total environmental impact were slightly higher for the MAE process that used the milled plant material.

However, since this process provided more TEM, the overall specific energy consumption per kg TEM and electricity cost per kg of TEM are lower, which reflects the higher TEM yield per kWh.

According to these preliminary results, it is suggested that future studies regarding the optimization of the MAE should involve using milled material, since milling enabled obtaining the extract with higher TEM values, which also showed that it possesses antimicrobial potential.

## 4. Conclusions

Under the tested laboratory conditions, the defined milling pretreatment of *V. album* plant material was associated with an MAE-produced extract with higher TEM values and with differences in antimicrobial performance. At the 20 min extraction plateau, TEM yield was 5.52 g/100 g plant material for the milled sample and 5.01 g/100 g for the non-milled sample. Qualitative UHPLC-DAD-MS/MS profiling showed organic acids, phenolic acids, flavonoid glycosides, and other secondary-metabolite derivatives. The MAE process that used the milled plant material had lower specific energy consumption relative to the TEM amount, together with a lower overall cost of the process. The extract from the milled material also showed improved antimicrobial potential. Therefore, *V. album* plant material from South Serbia should be explored further in terms of optimizing the MAE process, suggesting a focus on milling conditions as well. Further work should also include granulometric characterization, quantitative determination of isolated metabolites, and additional safety, stability, dose–response, and efficacy studies before specific applications are proposed.

## Figures and Tables

**Table 1:** TEM values and their percentage ratios relative to the highest overall TEM at different extraction times.

t (min)	TEM (g/100 g Plant Material)		R (%) ^a^	
	Milled Plant Material	Non-Milled Plant Material	Milled Plant Material	Non-Milled Plant Material
0	0	0	0	0
2	2.12 ± 0.006	2.00 ± 0.005	38.29	36.19
4	2.46 ± 0.005	2.25 ± 0.004	44.47	40.82
6	2.90 ± 0.004	2.79 ± 0.005	52.50	50.50
8	3.36 ± 0.004	3.03 ± 0.004	60.77	54.78
10	3.82 ± 0.004	3.46 ± 0.003	69.20	62.58
12	4.11 ± 0.003	3.90 ± 0.003	74.43	70.54
14	4.37 ± 0.003	4.01 ± 0.002	79.09	72.62
16	4.88 ± 0.003	4.36 ± 0.003	88.31	78.91
18	5.29 ± 0.001	4.81 ± 0.002	95.77	87.06
20	5.52 ± 0.001	5.01 ± 0.001	100.00	90.75
30	5.52 ± 0.001	5.01 ± 0.001	100.00	90.75

TEM values are presented as mean ± standard deviation of triplicate determinations. ^a^ R (%) was calculated using the highest overall TEM value observed across both plant-material preparations (5.52 g/100 g plant material) as the common denominator in Equation (2).

**Table 3:** Growth-inhibition zones (mm) produced by *V. album* extracts and reference antimicrobial agents.

Used Antimicrobial Agent	Microorganism							
	*B. cereus*	*B. subtilis*	*C. albicans*	*E. coli*	*K. pneumoniae*	*P. aeruginosa*	*P. vulgaris*	*S. aureus*
Milled plant material extract	29.33 ± 1.15	34.67 ± 1.15	21 ± 1	20.67 ± 0.58	26.33 ± 1.53	15.67 ± 0.58	21.33 ± 1.15	25 ± 1
Non-milled plant material extract	24.67 ± 1.15	30.67 ± 1.15	18.33 ± 0.58	18.33 ± 0.58	17.67 ± 0.58	NI	19.67 ± 0.58	24.33 ± 1.15
Nystatin	NT	NT	17 ± 1.3	NT	NT	NT	NT	NT
Ceftriaxone	22 ± 0.3	15 ± 0.3	NT	29 ± 1	20 ± 1	32 ± 1	30 ± 0.6	30 ± 0
Cefoxitin	20 ± 0.6	30 ± 0.6	NT	21 ± 0.6	25 ± 0.3	25 ± 0.3	32 ± 0.6	26 ± 1
Ciprofloxacin	32 ± 0.6	30 ± 0.3	NT	38 ± 0.6	31 ± 0.6	38 ± 1	24 ± 1	25 ± 1
Gentamicin	20 ± 1	23 ± 0.3	NT	22 ± 0.6	20 ± 0.3	20 ± 0.3	17 ± 0.3	23 ± 1
Imipenem	25 ± 0.6	37 ± 0.6	NT	25 ± 0.3	27 ± 0.3	20 ± 0.6	40 ± 1	39 ± 0.3

Values for *V. album* extracts are mean ± SD from three independent determinations. NI, no inhibition; NT, not tested.

**Table 4:** Minimum inhibitory concentrations (MICs) of *V. album* extracts against the tested microorganisms (µL/mL).

Used Antimicrobial Agent	Microorganism							
	*B. cereus*	*B. subtilis*	*C. albicans*	*E. coli*	*K. pneumoniae*	*P. aeruginosa*	*P. vulgaris*	*S. aureus*
Milled plant material extract	16	8	4	8	16	32	32	8
Non-milled plant material extract	32	16	8	16	32	32	32	16

**Table 5:** Comparative analysis of energy demand, environmental impact and cost efficiency of the proposed MAE methodologies.

Parameter	Unit	Milled Plant Material Extract	Non-Milled Plant Material Extract
Mass of plant material	g	100	100
Moisture content in plant material	wt.%	10.05	10.95
Solvent system volume	mL	1000	1000
Liquid-to-solid ratio	mL/g	10	10
Mass of water per mass of plant material	kg per kg	3	3
Mass of ethanol per mass of plant material	kg per kg	5.53	5.53
Mass of solvent system per mass of plant material	kg per kg	8.53	8.53
Temperature	°C	60	60
MAE power (W)	W	500	500
Mill power (W)	W	300	300
MAE time, min	min	20	20
Milling time, min	min	0.5	0
Total time consumption	min	20.5	20
MAE electricity consumption per batch	kWh	0.17	0.17
Milling electricity consumption per batch	kWh	0.0025	0
Total electricity consumption per batch	kWh	0.1725	0.17
MAE environmental impact	g CO_2_	133.3	133.3
Milling environmental impact	g CO_2_	2	0
Total environmental impact	g CO_2_	135.3	133.3
MAE energy input	kJ	600	600
Milling energy input	kJ	9	0
Total energy input	kJ	609	600
Thermal conductance	kW/K	150.1 · 10^−5^	150.1 · 10^−5^
Heat capacity	kWh/K	50.78 · 10^−5^	50.03 · 10^−5^
Specific heat capacity	kWh/K per kg per kg	5.95286 · 10^−5^	5.86489 · 10^−5^
TEM yield	g/g plant material	0.055	0.05
TEM extracted per batch	g	4.97	4.46
Specific energy consumption per kg dry matter	kJ/kg dry matter	6770.43	6737.8
Specific energy consumption per kg TEM	kJ/kg TEM	122,572.7	134,419.7
Electricity cost per batch *	€	0.049	0.0483
Electricity cost per kg of TEM *	€	9.86	10.81
TEM yield per kWh	g/g plant material/kWh	0.33	0.301
TEM yield per kWh/K	g/g plant material/kWh/K	98.01 · 10^−5^	90.3 · 10^−5^
TEM yield per kWh·K	g/g plant material/kWh·K	108.8	100.2

* The average electricity price in the EU for households of 0.2896 €/kWh (https://ec.europa.eu/eurostat/web/products-eurostat-news/w/ddn-20260505-1, accessed 19 October 2026).

## Data Availability

Data supporting the findings of this study are available from the corresponding author upon reasonable request.

## Supplementary Materials

The following supporting information can be downloaded at: https://www.mdpi.com/article/10.3390/plants15213311/s1, Table S1. Fragmentation details for the used reference standard compounds. Figure S1. MS/MS spectrum (upper panel) and UV–Vis absorption spectrum (lower panel) of compound 11 (Table 2), assigned as cryptochlorogenic acid. Figure S2. MS/MS spectrum (upper panel) and UV–Vis absorption spectrum (lower panel) of compound 28 (Table 2), assigned as 4′-hydroxy-5,7-dimethoxyflavanone-acetylpentosyl-pentose. Figure S3. MS/MS spectrum (upper panel) and UV–Vis absorption spectrum (lower panel) of compound 36 (Table 2), assigned as rhamnazin.

## Abbreviations

MAE	microwave-assisted extraction
TEM	total extractive matter
UHPLC-DAD-MS/MS	ultra-high-performance liquid chromatography with diode-array detection and tandem mass spectrometry
MIC	minimum inhibitory concentration
