# Supplementary material for: Qualitative Phytochemical Profiling and Antimicrobial Activity of Viscum album L. Extracts Obtained by Microwave-Assisted Extraction: Influence of Milling Pretreatment
Source: Plants (Basel). 2026 Oct 26;15(21):3311. doi: 10.3390/plants15213311 (PMC13765698; doi:10.3390/plants15213311)
Supplement: Supplementary file 1 [file plants-15-03311-s001.zip › plants-4615657-supplementary.pdf]

SUPPLEMENTARY MATERIAL

Table S1. Fragmentation details for the used reference standard compounds

Standard	t_R, min	λ_{max}, nm	$[M\text{-}H]^-$, *m/z*	MS/MS fragment ions
Apigenin	7.62	267;338	269	225,201,197,183,151,149,117
Caffeic acid	5.54	298sh;325	179	143,135(100%),71
Chlorogenic acid	5.13	300sh;326	353	191(100%)
Citric acid	0.92	–	191	173,111(100%),99,85
Hyperoside	8.82	258;356	463	300/301(100%),271,255,179,151
Isoquercitrin	8.87	258;356	463	300/301(100%),271,255,179,151
Kaempferol	12.05	266,366	285	285(100%),257,241,229,151,133,117,93
Luteolin	11.35	262;350	285	285(100%),241,217,199,175,151
Malic acid	0.83	–	133	115(100%),87
Neochlorogenic acid	2.33	297sh;325	353	191(100%),179,173,135
Quercetin	11.13	257;371	301	273,257,245,229,193,179(100%),175,165,151,121,107
Rosmarinic acid	9.69	290;330	359	197,179,161(100%),135
Rutin	8.98	258,355	609	591,463,343,300/301(100%),271,255,228
Tiliroside	11.49	268;317	593	447,307,285(100%),255,227,163,145,119

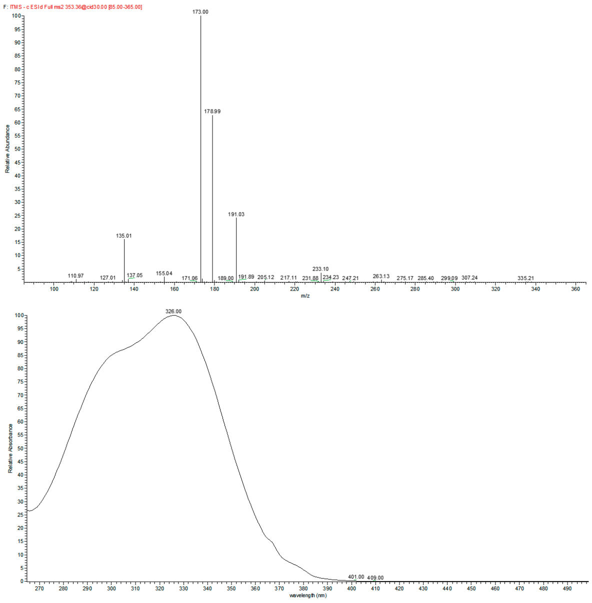

Figure S1. MS/MS spectrum (upper panel) and UV–Vis absorption spectrum (lower panel) of compound 11 (Table 2), assigned as cryptochlorogenic acid.

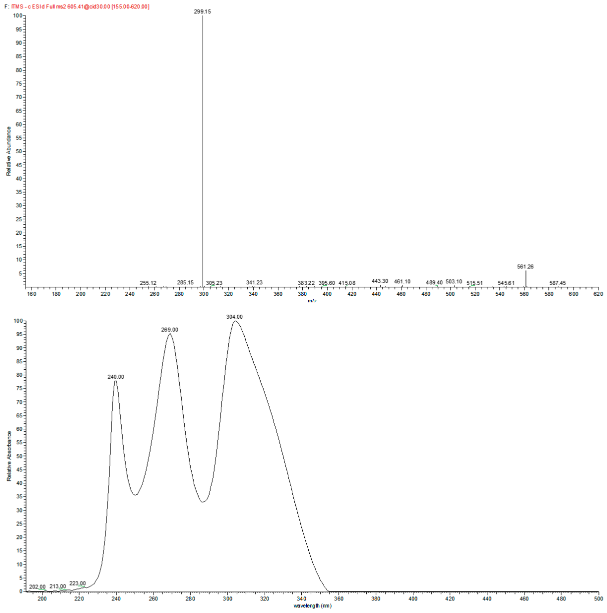

Figure S2. MS/MS spectrum (upper panel) and UV–Vis absorption spectrum (lower panel) of compound 28 (Table 2), assigned as 4′-hydroxy-5,7-dimethoxyflavanone-acetylpentosyl-pentose.

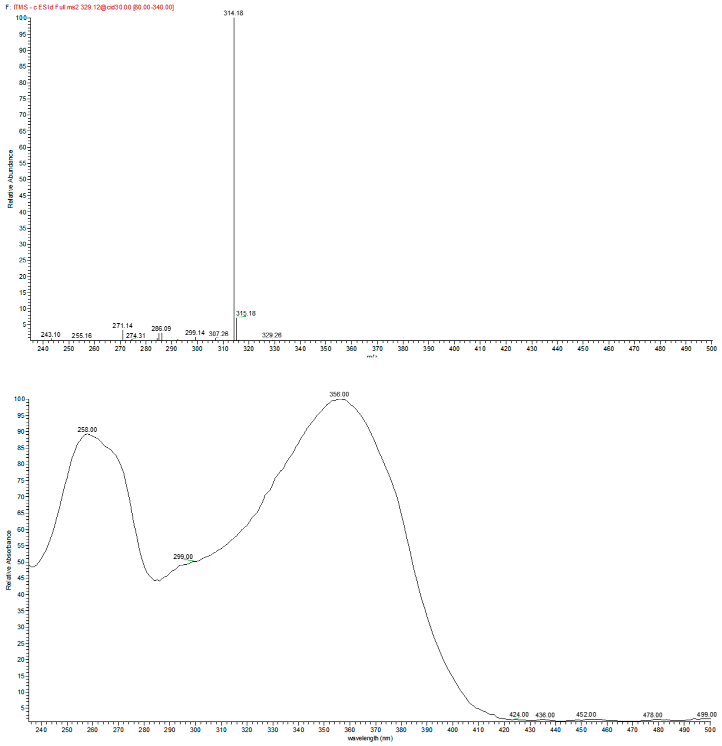

Figure S3. MS/MS spectrum (upper panel) and UV–Vis absorption spectrum (lower panel) of compound 36 (Table 2), assigned as rhamnazin.
